# Aminoglycoside-induced nephrotoxicity in children

**DOI:** 10.1007/s00467-016-3533-z

**Published:** 2016-11-15

**Authors:** Stephen J McWilliam, Daniel J Antoine, Rosalind L Smyth, Munir Pirmohamed

**Affiliations:** 1 Department of Women’s and Children’s Health, University of Liverpool, Liverpool, UK; 2 Department of Molecular and Clinical Pharmacology, University of Liverpool, Liverpool, UK; 3 Institute of Child Health, University College London, London, UK

**Keywords:** Aminoglycoside, Nephrotoxicity, Acute kidney injury, Biomarker, Kidney injury molecule-1

## Abstract

Aminoglycoside antibiotics, in particular gentamicin and tobramycin, are still commonly used in paediatric clinical practice. These drugs cause nephrotoxicity, which particularly affects the proximal tubule epithelial cells due to selective endocytosis and accumulation of aminoglycosides via the multi-ligand receptor megalin. Recent epidemiological studies, using more widely accepted definitions of acute kidney injury (AKI), have suggested that AKI may occur in between 20 and 33 % of children exposed to aminoglycosides. A consensus set of phenotypic criteria for aminoglycoside-induced nephrotoxicity have recently been published. These are specifically designed to provide robust phenotyping for pharmacogenomic studies, but they can pave the way for standardisation for all clinical studies. Novel renal biomarkers, in particular kidney injury molecule-1, identify aminoglycoside-induced proximal tubular injury earlier than traditional markers and have shown promise in observational studies. Further studies need to demonstrate a clear association with clinically relevant outcomes to inform translation into clinical practice. Extended interval dosing of aminoglycosides results in a reduction in nephrotoxicity, but its use needs to become more widespread. Inhibition of megalin-mediated endocytosis by statins represents a novel approach to the prevention of aminoglycoside-induced nephrotoxicity which is currently being evaluated in a clinical trial. Recommendations for future directions are provided.

## Introduction

The first aminoglycoside, streptomycin, was introduced into clinical practice in 1944, and has since been followed by many drugs of this class [1]. The most common of these in clinical practice are gentamicin and tobramycin. The aminoglycosides are particularly active against aerobic Gram-negative bacteria, including Enterobacteriaceae and *Pseudomonas*. Clinically, aminoglycosides may be used to provide targeted therapy, such as for the treatment of pulmonary exacerbations in children with cystic fibrosis colonised with *Pseudomonas aeruginosa*. They are also used for the empirical treatment of suspected systemic sepsis, where they are given in combination with other antibiotics (such as gylcopeptide or beta-lactam antibiotics) to provide broad spectrum coverage of Gram-positive and Gram-negative bacterial species.

Nephrotoxicity, one of the most important adverse effects linked to aminoglycoside exposure, is associated with a varying degree of renal tubular dysfunction that may in the most severely affected patients lead to non-oliguric acute kidney injury (AKI) [1]. This review has three aims: first, to evaluate what is currently known about aminoglycoside nephrotoxicity in children; second, to describe recent advances in the field, including novel diagnostics and therapeutic approaches; third, to provide recommendations on future directions for research.

## Mechanisms of aminoglycoside-induced nephrotoxicity

Aminoglycoside-induced nephrotoxicity is characterised by selective targeting of the proximal tubule epithelial cells within the renal cortex. Approximately 5 % of the administered dose accumulates within these cells after glomerular filtration [2]. Endocytosis via the multi-ligand receptor megalin has been demonstrated to be the principal pathway for this accumulation: megalin knock-out mice do not exhibit renal accumulation of aminoglycosides [3]. Megalin is a ligand for numerous low-molecular-weight proteins (including albumin, vitamin D-binding protein, retinol-binding protein, α1-microglobulin and β2-microglobulin) and is highly expressed by proximal tubule epithelial cells [4, 5], explaining the cell- and tissue-specificity of this toxicity.

Once inside the cell, aminoglycosides accumulate within lysosomes [6], the Golgi apparatus and endoplasmic reticulum (ER) [7], binding to phospholipids and inhibiting phospholipase activity, which results in lysosomal phospholipidosis [6, 8]. At some unknown threshold concentration of aminoglycoside, leakage occurs from the lysosomal structures into the cytoplasm [9]. Cytoplasmic aminoglycoside then acts both directly and indirectly on the mitochondria, activating the intrinsic pathway of apoptosis via cytochrome *c* [10] which in turn leads to the disruption of electron transport and ATP production and the formation of reactive oxygen species [8]. Lysosomal cathepsins, released into the cytoplasm, also activate the intrinsic apoptotic pathway [11] and, in higher concentrations, may cause necrosis [12]. In the ER, aminoglycosides inhibit protein synthesis and associated ER functions, resulting in ER stress and apoptosis via calpain and caspase 12 [13].

The reasons for inter-individual variability in susceptibility to aminoglycoside-induced nephrotoxicity are not clear. In particular, it is not known whether there are genetic factors which increase susceptibility, as has been reported for aminoglycoside-related hearing loss [14]. No genome-wide studies have been undertaken in this area.

It can be hypothesised from the literature that mutations resulting in megalin deficiency would be protective, as in megalin knock-out mice [3]. Other proteins also play a role in the pathway of megalin-mediated endocytosis. For example, the CIC-5 protein, which is defective in Dent’s disease, is involved in megalin trafficking [15]. In their study on renal accumulation of aminoglycoside in CIC-5 knockout mice compared to controls, Raggi et al. observed that there was an 85 % reduction in gentamicin accumulation in the knockout mice [15]. The same group also demonstrated a 15 % decrease in gentamicin accumulation in mice with defective *CFTR*, the gene affected in cystic fibrosis, and hypothesised that CFTR may play a role in the pathway of megalin-mediated endocytosis [15]. These results suggest that a genetic variant which impairs the megalin-mediated uptake pathway would therefore also provide some protection against aminoglycoside-induced nephrotoxicity. However, no human studies have as yet investigated this proposal.

## Defining aminoglycoside-induced nephrotoxicity

A key difficulty in establishing the epidemiology of drug-induced kidney injury, including that caused by aminoglycosides, has been the absence of consensus criteria for diagnosing kidney damage. There are a number of classification systems for classifying AKI in children, of which the best validated are the paediatric-modified RIFLE (pRIFLE) criteria [16, 17] and the Acute Kidney Injury Network (AKIN) criteria [18]. The pRIFLE criteria depend on using estimated creatinine clearance, while the AKIN criteria are based on measured serum creatinine (Table 1). pRIFLE criteria seem to be the more sensitive of the two classification systems, but the AKIN criteria have a stronger association with poor outcomes, suggesting that they may be more specific [19, 20]. More recently, the Kidney Disease: Improving Global Outcomes (KDIGO) clinical practice guideline for AKI [21] has attempted to develop consensus (Table 1).

**Table 1:** Paediatric acute kidney injury definitions

Paediatric Risk, Injury, Failure, Loss, End-Stage Kidney Disease (pRIFLE) system			Acute Kidney Injury Network (AKIN) guideline			Kidney Disease: Improving Global Outcomes (KDIGO) guideline		
AKI severity	Estimated creatinine clearance	Urine output	AKI severity	Serum creatinine	Urine output	AKI severity	Serum creatinine	Urine output
‘Risk’ (R)	Decrease by 25 %	<0.5 ml/kg/h for 8 h	Stage 1	≥0.3 mg/dl (26.5 μmol/L) rise OR Increase to 1.5–1.99× baseline	<0.5 ml/kg/h for >6 h	Stage 1	1.5–1.9× baseline OR ≥0.3 mg/dl (≥26.5 μmol/l) increase	<0.5 ml/kg/h for 6–12 h
‘Injury’ (I)	Decrease by 50 %	<0.5 ml/kg/h for 16 h	Stage 2	Rise to ≥2–2.99× baseline	<0.5 ml/kg/h for >12 h	Stage 2	2.0–2.9× baseline	<0.5 ml/kg/h for ≥12 h
‘Failure’ (F)	Decrease by 75 % OR Creatinine clearance of <35 ml/min/1.73 m^2^	<0.3 ml/kg/h for 24 h OR anuria for 12 h	Stage 3	Rise to ≥3× baseline OR ≥4 mg/dl (353.6 μmol/L) rise with an acute rise of at least 0.5 mg/dl (44 μmol/L)	<0.3 ml/kg/h for 24 h OR Anuria for 12 h	Stage 3	3.0× baseline OR Increase in serum creatinine to ≥4.0 mg/dl (≥353.6 μmol/l) OR Initiation of renal replacement therapy OR In patients aged <18 years, decrease in estimated glomerular filtration rate to <35 ml/min per 1.73 m^2^	<0.3 ml/kg/h for ≥24 h OR Anuria for ≥12 h

The paediatric acute kidney injury definitions presented in this table are adapted from Akcan-Arika [16] and Kellum et al. [21]

A standardised set of phenotypic criteria for drug-induced kidney disease (DIKD) has also recently been published by Mehta et al. [22]. This work was initiated by the International Serious Adverse Event Consortium, following on from previous work by this group in developing phenotypic criteria for other drug-induced adverse events [23]. Although the initial purpose of these criteria is to provide clear phenotypes for genetic studies of DIKD, they may also provide a consistent framework for all research in DIKD. Four phenotypes of DIKD have been proposed:

- AKI
- Glomerular disorder
- Tubular disorder
- Nephrolithiasis/crystalluria

In relation to aminoglycoside-induced nephrotoxicity, the authors have suggested that it should be characterised by the AKI phenotype. The criteria for this phenotype are summarised in Table 2. An issue which also needs to be considered in a child with AKI is causality, i.e. whether the AKI is due to the infection for which the aminoglycoside was prescribed, or due to the drug per se. As there are no specific diagnostic tests for aminoglycoside nephrotoxicity, standardised causality assessment tools should be used, of which many have been described [24].

**Table 2:** Suggested phenotypic criteria for drug-induced acute kidney injury, including that caused by aminoglycosides

Primary criteria	Secondary criteria
• Rise in serum creatinine that presents as or progresses to stage 2 (KDIGO) 2–2.9× reference serum creatinine or higher • If child has a baseline serum creatinine of <0.5 mg/dl (44 μmol/L), must double serum creatinine to get to at least 0.5 mg/dl (44 μmol/L) or above OR • Decline by at least 50 % from peak serum creatinine over 7 days in relationship to change in drug-dosing adjustment or discontinuation within 2 weeks	• Oliguric <0.5 ml/kg per hour for 12 h (KDIGO stage 2) • Non-oliguric >1 ml/kg per hour for 24 h (paediatrics) • Urinalysis findings: granular and muddy casts consistent with acute tubular necrosis, urinary eosinophils, proteinuria • Fractional excretion of sodium of >1 % • Negative ultrasound findings • Positive gallium scan for acute interstitial nephritis • Clinical symptoms for acute interstitial nephritis: fever, rash and joint pains

The phenotypic criteria for drug-induced acute kidney injury presented in this table are adapted from Mehta et al. [22]

## Epidemiology of aminoglycoside-induced nephrotoxicity in children

### Children

Until recently, little data have been available documenting the incidence of aminoglycoside-induced nephrotoxicity in children, in part due to the lack of an accepted definition for AKI. However, following the recent advent of more widely accepted definitions of AKI in children, a number of studies have begun to address this question.

A retrospective cohort study at a tertiary children’s hospital in the USA used the pRIFLE criteria and the AKIN Staging definition to define aminoglycoside-induced nephrotoxicity [19]. Of the 557 children who received aminoglycoside treatment for ≥5 days over the year of the study, the AKI rate was 33 and 20 % using the pRIFLE and AKIN criteria, respectively. AKI was associated with longer hospital stay and higher total hospital costs. The authors reported that whilst pRIFLE was more sensitive for the identification of AKI, AKIN was more strongly associated with patient outcomes.

Another factor limiting the quality of epidemiological data available is inconsistency among monitoring practices. In a prospective study of all admissions involving aminoglycoside exposure for ≥3 days at a tertiary paediatric centre in the USA, daily measurement of serum creatinine was used to monitor for AKI. AKI, defined by the pRIFLE criteria, was identified in 25 % of unique patients exposed to aminoglycosides and during 31 % of admission episodes [25].

### Neonates

Gentamicin is the drug most commonly prescribed to neonates in the UK [26]. An American study found that 57.5 % of all neonates discharged from the neonate intensive care unit had received treatment with gentamicin [27]. The UK National Institute of Health and Care Excellence (NICE) guidelines recommend the use of gentamicin (in combination with a penicillin) for first line therapy in neonates with suspected early-onset sepsis [28]. Despite its widespread use, there are a paucity of data quantifying gentamicin-induced nephrotoxicity in neonates [29].

A retrospective study of nephrotoxin exposure in preterm neonates at one U.S. centre documented gentamicin exposure in 86.0 % of the 107 neonates [30]. In this cohort, 26.2 % developed AKI. Whilst this study cannot demonstrate causation, it does highlight the potential adverse impact of exposure to nephrotoxins in this population. A recent narrative review included ten studies of gentamicin use in neonates, where nephrotoxicity was assessed using plasma creatinine [31]. Interestingly, seven of these studies reported no nephrotoxicity, while the remaining three reported various rates, the maximum being 27 % [32]. This wide variation in rates again highlights the previous lack of standardised criteria for diagnosing aminoglycoside nephrotoxicity.

### Children with cystic fibrosis

Cystic fibrosis (CF) is an autosomal recessive life-limiting disease which affects around 9000 people in the UK alone [33]. The disease is characterised by the accumulation of thick secretions in the airways of the lungs that lead to reduced activity of the mucociliary escalator and predispose to secondary bacterial infection and pulmonary colonisation, often by resistant organisms, in particular *Pseudomonas aeruginosa*. Approximately 25 % of children with CF aged 12–15 years have chronic pulmonary infection with *P. aeruginosa*; this infection rate rises to 40 % by 16–19 years of age [33].

Aminoglycosides have good efficacy against *P. aeruginosa* and are commonly used intravenously to treat pulmonary exacerbations in CF in combination with a beta-lactam antibiotic, such as ceftazidime. Treatment courses usually last for 2 weeks, and patients may have multiple courses of treatment throughout their lifetime.

A UK national survey of AKI in patients with CF found 24 cases between 1997 and 2004 [34]; of these 88 % of patients were receiving an aminoglycoside at the time of developing AKI, or within the previous week. Identification of AKI relied on physician report and did not use standardised criteria—rather the AKI was defined as ‘raised plasma creatinine for age with or without oliguria’ [34]. A follow-on case–control study identified an 80-fold increase in the risk of AKI if CF patients received an aminoglycoside within the preceding week [35]. AKI was associated with significant acute morbidity, with 54 % requiring dialysis [34].

The impact of daily monitoring of serum creatinine during treatment with aminoglycosides in children with CF has been assessed in a retrospective study in a tertiary paediatric centre in the USA [36]. AKI was defined as a rise in serum creatinine by ≥0.3 mg/dl (26.5 μmol/L) within 48 h, or a 1.5-fold increase in the baseline serum creatinine level. Daily monitoring not only led to more cases of AKI being identified (in 21 of 103 courses, 20 %), but also an earlier identification of AKI. The authors of this study suggested that daily monitoring also led to changes in management (including increased use of once-daily dosing of aminoglycosides and intravenous (IV) fluids, reduced use of concomitant nephrotoxins and shorter courses of aminoglycosides) in an attempt to prevent or ameliorate AKI, although a randomised trial would be required to assess whether there was any impact on patient outcomes. In a second study, which had a case–control design, the same group identified that of the 593 admissions in which children were treated with an aminoglycoside for an exacerbation of CF [37], there were 82 cases of AKI (14 %) which they felt were aminoglycoside-induced.

## Long-term outcomes of aminoglycoside-induced nephrotoxicity

Nephrotoxin-associated AKI may lead to chronic kidney disease (CKD) [38]. In a retrospective cohort study, children who developed AKI (using pRIFLE criteria) associated with nephrotoxin exposure (≥3 days of aminoglycosides or ≥3 nephrotoxins simultaneously for 1 day) had a relative risk of 3.84 [95 % confidence interval (CI) 1.57–9.40, *P* <0.05] for developing one or more signs of CKD [reduced estimated glomerular filtration rate (GFR), hyperfiltration, proteinuria, or hypertension] at 6 months compared to controls (nephrotoxin exposure, but no AKI) [38].

The long-term effects of multiple exposures to aminoglycosides are less clear. In a cohort of adults with CF from Liverpool, chronic renal impairment (as measured by reduced creatinine clearance) was reported in 31–42 % of adult CF patients (depending on definition) and was associated with cumulative aminoglycoside exposure (*P*= 0.0055) [39]. This effect was exacerbated by the concomitant use of intravenous (IV) colistin, although IV colistin alone did not have an impact on renal function [39]. A previous, slightly smaller study in a Danish CF centre using tobramycin found no association between previous tobramycin exposure and measured creatinine clearance [40].

## Biomarkers of aminoglycoside-induced nephrotoxicity

### Existing approaches

The traditional indicator of AKI is a rise in serum creatinine concentration, which forms the basis of all current AKI definitions (Table 1). A rise in serum creatinine concentration as an indicator of GFR has long been established in clinical practice and is simple to measure; in addition, the test is very widely available. However, an elevation of serum creatinine is a delayed response, with levels rising significantly above baseline only when 25–50 % of renal function has been lost [41]. Reliance upon this measurement means that AKI is frequently not identified early and that the degree of damage may be underestimated [42]. Furthermore, an increased level of serum creatinine is a marker of glomerular filtration and not an indicator of damage at other sites in the nephron. Interpretation is made more difficult by the variation in the production of creatinine, which depends on age, sex and weight (in particular muscle mass). Interpretation is also difficult in the newborn, in whom serum creatinine initially reflects maternal values.

### Potential of novel biomarkers

Early identification of nephrotoxicity requires a sensitive marker that can be quantified earlier than is the case with currently used indicators. Identification of aminoglycoside-induced nephrotoxicity also requires a biomarker that is specific for the resulting proximal tubule injury, which would allow for early treatment adjustment, intervention and the avoidance of further injury.

The Predictive Safety Testing Consortium (PSTC), a collaboration of academic, industry (both pharmaceutical and biotechnology companies) and regulatory [the US Food and Drug Agency (FDA) and the European Medicines Agency (EMA)] partners, was established to expedite the qualification of renal biomarkers of nephrotoxicity. The work of the PSTC has led to the qualification of seven renal biomarkers for pre-clinical use by the FDA, EMA and Japanese Pharmaceuticals and Medical Devices Agency (PMDA), namely, kidney injury molecule-1 (KIM-1), albumin, total protein, β2-microglobulin, cystatin C, clusterin and trefoil factor-3 [43].

Amongst those approved by the FDA, EMA and PMDA, only KIM-1 is specific for the proximal tubule. Albumin, β2-microglobulin, and cystatin C may reflect damage to both the proximal tubule and the glomerulus, whereas clusterin may reflect damage to both the proximal and distal tubule [44]. Total protein reflects glomerular injury [44], and trefoil factor-3 is expressed in the collecting duct [45]. The albumin/creatinine ratio is widely utilised clinically as a marker of renal disease and response to treatment. However, its urinary concentration may be altered by both changes in glomerular permeability and tubular reabsorption. Furthermore, the concentration of albumin may be increased by additional factors, including fever, exercise, dehydration, diabetes and hypertension, thereby limiting its specificity for AKI and, in particular, for aminoglycoside-induced nephrotoxicity [44]. Of those renal biomarkers which have not been qualified through the work of the PSTC, neutrophil gelatinase-associated lipocalin (NGAL) and *N*-acetyl-β-D-glucosaminidase (NAG) have received the most interest as biomarkers of proximal tubular injury. The potential of KIM-1, NGAL and NAG as biomarkers of aminoglycoside-induced nephrotoxicity is detailed in Table 3.

**Table 3:** Comparative description of three novel urinary biomarkers and their utility in aminoglycoside-induced nephrotoxicity

Biomarker	Description	Utility in aminoglycoside-induced nephrotoxicity	Comments
Kidney Injury Molecule-1 (KIM-1)	Cell membrane glycoprotein upregulated by proximal tubule epithelial cells in response to toxicity [73] Confers a phagocytic phenotype [74]	Outperforms, with respect to sensitivity and specificity, traditional and novel biomarkers of AKI (serum creatinine, blood urea nitrogen, and NAG), as confirmed by histopathology in animal models [46] Early diagnostic marker for AKI and predictor of mortality risk [75] Elevated during aminoglycoside exposure in preterm neonates [47] and children with CF [49, 50]	Specific to proximal tubule Outperforms other biomarkers in pre-clinical models of aminoglycoside-induced nephrotoxicity
Neutrophil Gelatinase-associated Lipocalin (NGAL)	25-kDa protein expressed by kidney epithelial cells (and other tissues, as well as neutrophils) [76]	Upregulated in response to nephrotoxins in mouse models [77] Sensitive early predictor for AKI [75] Elevated during aminoglycoside exposure in preterm neonates [47]	Levels elevated in sepsis/inflammation [78] which may limit specificity
N-acetyl-β-D-glucosaminidase (NAG)	130- to 140-kDa lysosomal enzyme specific to proximal tubule epithelial cells [79]	Widely used in pre-clinical and clinical studies of aminoglycoside-induced nephrotoxicity [80] Elevated during aminoglycoside exposure in preterm neonates [47]	Outperformed by KIM-1 in pre-clinical models of aminoglycoside-induced nephrotoxicity

AKI, Acute kidney injury; CF, cystic fibrosis

In line with preclinical data [46], KIM-1 outperformed other biomarkers (NGAL, NAG and serum creatinine) in the identification of the potential for aminoglycoside-induced nephrotoxicity in preterm neonates [47]. In children with CF, urinary KIM-1 concentration was significantly correlated with the number of previous courses of aminoglycosides (*r*= 0.35, *P* = 0.012) [48]. Our group has replicated this finding in a UK paediatric CF cohort (*R*= 0.70, *P* *<*0.002), published in abstract form [49]. This work also identified acute elevation in KIM-1 concentrations during aminoglycoside exposure [49], which has also been reported elsewhere in abstract form [50].

## Preventing aminoglycoside-induced nephrotoxicity

### Existing approaches

#### Choice of aminoglycoside

The choice of aminoglycoside is important in reducing nephrotoxicity. The following rank order of nephrotoxicity has been reported, from most toxic to least toxic: neomycin > gentamicin ≥ tobramycin ≥ amikacin ≥ netilmicin > streptomycin [1]. However, it is noted that the differences from gentamicin through to netilmicin may be small and that the results in clinical trials are inconsistent.

A meta-analysis of 43 randomised trials in both adults and children comparing the efficacy and toxicity of aminoglycosides calculated pooled odds ratios for the potential of the different drugs to cause nephrotoxicity [51]. A weakness of this approach is that the studies used different definitions of nephrotoxicity, which brings into question the validity of combining different studies. However, the authors of the meta-analysis reported that tobramycin demonstrated less nephrotoxicity than gentamicin (odds ratio 0.64, 95 % CI 0.42–0.97).

In a case–control study of paediatric and adult patients with CF, gentamicin exposure in the previous year was associated with AKI (19/24 cases vs. 1/42 controls; *P* < 0.001, odds ratio incalculable), whereas tobramycin was not (9/24 cases vs. 16/42 controls, *P* = 0.9, odds ratio 1.0, 95 % CI 0.3–2.6) [35]. Moreover, gentamicin resistance has been reported in the majority of *P. aeruginosa* isolates from CF patients [52]. There has therefore been a shift away from using gentamicin and towards tobramycin, to which *P. aeruginosa* is more widely sensitive, in the management of acute exacerbations of CF.

#### Extended interval dosing

In theory, extended interval dosing of aminoglycosides may be safer for the kidneys. A higher single dose may result in saturation of megalin-mediated uptake of aminoglycoside in the proximal tubule, resulting in a greater percentage of the aminoglycoside being excreted in the urine [53]. The TOPIC study was a well-powered clinical trial comparing one versus three daily doses of IV tobramycin for pulmonary exacerbations in adults and children with CF [54]. The authors of this study demonstrated an equivalent efficacy of both regimens using the change in forced expiratory volume in 1 s (FEV1) as their primary outcome measure. In children, there was a 3.7 % increase in serum creatinine with three daily doses compared with a 4.5 % decrease with a single daily dose of tobramycin, a difference which was statistically significant. This finding was supported by measurements of NAG pre-treatment and after 14 days of tobramycin: the increase in urinary NAG was 33 % less with once-daily dosing compared to the three daily doses administered to the treatment group (*P* = 0.049 in adults and children, and *P* = 0.02 in children alone). With evidence of equal efficacy, and reduced nephrotoxicity, this study has led to widespread adoption of once-daily dosing of tobramycin (10 mg/kg) and a move away from dosing three times daily in children with CF. However, the uptake of extended interval dosing is not universal: a recent survey in the USA reported that extended interval dosing of aminoglycosides was used in 63 % of hospitals [55].

A meta-analysis of studies comparing once-daily with multiple-daily doses of aminoglycosides in children has also been undertaken [56]. However, definitions of toxicity were not consistent across the studies. The authors of the meta-analysis defined primary nephrotoxicity as ‘a rise in serum creatinine or decrease in creatinine clearance with thresholds as defined in each study’. They found that 1.6 % of children experienced primary nephrotoxicity in both the once-daily and multiple-daily dosing groups [56].

#### Therapeutic drug monitoring

Therapeutic drug monitoring is felt to be helpful for monitoring both efficacy and toxicity. Elevated trough levels suggest reduced renal clearance of aminoglycoside. This may reflect pre-existing renal dysfunction, but is also considered to be a risk factor for developing nephrotoxicity: for example, several adults and children with CF who developed AKI had trough tobramycin levels above 2 mg/L [34]. There is no consistent guidance on the optimal regimen for therapeutic drug monitoring in either multiple-daily or extended interval dosing, and practice thus varies widely. For instance, UK CF guidelines recommend that levels measured 18 h after the previous dose (with a single daily dose) should be <1 mg/L [57]. U.S. CF guidance recommends levels taken between 9 and 11 hour post-dose should be undetectable [58]. With a peak, and another level 6–8 h post-dose, it is possible to use pharmacokinetic principles to adjust dosing to a target area under the curve (AUC) [59]. This approach is used in some centres in both adults and children, but there is little evidence to define what the optimum AUC should be to maximise the benefit–risk ratio [59].

#### Nebulised aminoglycosides

Nebulised aminoglycosides (especially tobramycin) have become increasingly widely used in CF. They tend to be used over the long term in those patients with *P. aeruginosa* resulting in an improvement in FEV1 and a reduction in time spent in hospital in both adults and children [60]. The perceived benefits are that the antibiotic is delivered directly to the desired site of action and that it is therefore felt to be possible to deliver high local concentrations, with reduced systemic exposure. However, inhaled tobramycin is systemically absorbed [61], and AKI associated with nebulised tobramycin has been reported [62]. The incidence of nephrotoxicity is thought to be lower than that with IV aminoglycosides, but this has not been investigated in detail.

### Novel approaches

#### Daily monitoring of serum creatinine

In the NINJA study (Nephrotoxic Injury Negated by Just-in-time Action) systematic screening of electronic health records was instituted to identify children receiving IV aminoglycosides for ≥3 days or ≥3 simultaneous nephrotoxins. In the patients enrolled in this study, daily monitoring of serum creatinine was recommended. The mean weekly AKI rate was 25.5 % for nephrotoxin-exposed patients using the pRIFLE criteria [25]. A 42 % reduction in AKI intensity (from 33.6 to 19.5 days/100 exposure days) was reported during 1 year of implementation of the screening programme [25]. The results of a follow-up analysis of this project after 3 years of implementation revealed that there had been a 38 % reduction in exposure to nephrotoxic medications (11.63 to 7.24 exposures/1000 patient days), and a 64 % reduction in the AKI rate (2.96 to 1.06 episodes/1000 patient days) [63].

#### Time of dosing

A recently published, open label, randomised controlled trial (RCT) investigated the impact of time of day on the pharmacokinetics and nephrotoxicity of IV tobramycin in 18 children with CF [64]. Children received IV tobramycin at either 0800 or 2000 hours. Nephrotoxicity was assessed using a panel of urinary biomarkers, including KIM-1, cystatin C, NGAL, interleukin-18 and NAG. Urine was collected at baseline and at the end of a 14-day treatment course. There was no difference in renal clearance between the two groups. Of all the biomarkers measured, only KIM-1 was different between the two groups, with a significantly greater increase in the evening group compared to the morning group (*P* < 0.05), suggesting an increased risk of nephrotoxicity with evening administration. The authors postulated that this difference was due to the impact of established diurnal rhythms on the pharmacokinetics and pharmacodynamics of tobramycin, but this hypothesis needs further investigation. The possibility that morning administration of aminoglycosides may be safer than evening administration deserves further investigation in a larger RCT.

#### Pharmacological interventions

A large number of drugs have been proposed to have the potential to inhibit aminoglycoside-induced nephrotoxicity [65]. However, we are only aware of two which have been investigated in humans.

One small RCT assessed the calcium channel blocker nifedipine in adult patients receiving gentamicin for the treatment of upper urinary tract infection [66]. It was a small study, with a total of 32 participants. The primary outcome measure was change in creatinine clearance, and the results demonstrated an improvement in creatinine clearance in the intervention group, with a deterioration in the placebo group. Unfortunately, the trial had methodological limitations, and it is difficult to draw conclusions as to whether nifedipine had a true positive impact. Indeed, it is possible that the effect on the GFR was a direct effect of the nifedipine itself, secondary to vasodilation of the afferent arteriole, leading to increased glomerular perfusion, rather than due to any inhibition of gentamicin-induced nephrotoxicity. To our knowledge, there have been no further studies of calcium channel blockers in humans, while animal studies have not consistently shown that aminoglycoside-induced nephrotoxicity can be prevented by calcium-channel blockers [65].

A randomised crossover study of fosfomycin, an infrequently used broad-spectrum phosphonic acid antibiotic, in eight adult patients with CF receiving tobramycin and colistin showed that there was a small reduction in proteinuria with concomitant fosfomycin treatment; however, there was no effect on serum creatinine [67]. The size of this study is a limitation, and it has only been published in abstract form.

#### Inhibition of megalin-mediated endocytosis of aminoglycosides

Inhibition of megalin-mediated endocytosis, the primary pathway for the renal accumulation of aminoglycosides, has been proposed as a potential preventive strategy. Indeed, known megalin substrates, including cytochrome *c* and cationic peptide fragments, have demonstrated dose-dependent competitive inhibition of megalin-mediated uptake of gentamicin in vitro and inhibition of nephrotoxicity in vivo (rat and mouse) [68]. However, blockade of megalin with endogenous peptides does not provide a viable therapeutic strategy in humans as such peptides may be unstable and would need to be administered parenterally; in addition, there may be a risk of immunogenicity.

More promising are the results showing that statins can inhibit megalin-mediated endocytosis in vitro [69, 70]. In keeping with this, statins (simvastatin, pravastatin and rosuvastatin) inhibited gentamicin accumulation and cytotoxicity in a dose-dependent manner in an in vitro proximal tubule model [5]. Inhibition of gentamicin-induced nephrotoxicity by simvastatin has also been demonstrated in rats [71, 72].

We are currently investigating this mechanism in humans for the first time through a phase IIa randomised, controlled, clinical trial of rosuvastatin for the prevention of aminoglycoside-induced nephrotoxicity in children with CF (The PROteKT study; EudraCT 2014-002387-32, UKCRN ID 16993, ISRCTN26104255).

## Future directions

It is only recently, with the advent of standardised definitions of AKI, that studies have begun to shed more light on the epidemiology of aminoglycoside-induced nephrotoxicity. Large observational cohort studies of both paediatric and adult patients being treated with aminoglycosides are needed. However, the design of such studies is important. First, these would need to use a standardised definition of AKI [such as the KDIGO guideline (Table 1)] alongside standardised phenotypic criteria for aminoglycoside-induced nephrotoxicity (Table 2) [22]. Second, they should include baseline and serial measurements of novel renal biomarkers, in particular KIM-1, in order to assess the predictive value of these markers for AKI. Third, DNA should be collected for pharmacogenomic analyses. This approach will allow for the description of the size of the problem using accepted definitions, an assessment of the potential clinical utility of novel biomarkers and the identification of risk factors for the development of aminoglycoside-induced nephrotoxicity, including genetic factors.

The potential of novel, non-invasive, urinary biomarkers has been described. KIM-1 shows promise as a biomarker of acute and chronic proximal tubular injury associated with exposure to aminoglycosides [47–50] and outperforms other biomarkers in pre-clinical studies [46]. As described above, demonstrating a clear association with clinically relevant outcomes will inform future translation into clinical practice. Following this, further qualification of KIM-1, or any other promising biomarker, would be required through novel study designs (including RCT) where the measured biomarker concentrations are used to guide treatment decisions in patients exposed to aminoglycosides.

The development of improved diagnostic tools must be coupled with the development of further strategies to minimise the nephrotoxic consequences of aminoglycosides. A promising preventive strategy utilising statins has already been described and is currently being assessed in children with CF, using KIM-1 as the primary outcome measure. If successful, this intervention will require assessment in a large, phase III RCT using a standardised definition of AKI as the primary outcome measure in order to pave the way for widespread clinical application.

## Conclusion

Clinicians considering the use of aminoglycosides for the treatment of infection in their patients will be well aware of the potential for nephrotoxicity to occur with these antibiotics. Despite changes to practice, such as extended interval dosing, nephrotoxicity still occurs. Novel renal biomarkers, in particular KIM-1, may lead to the earlier identification of nephrotoxicity, ultimately allowing for timely intervention to prevent further kidney injury. Preventive strategies may ultimately lead to further changes in clinical practice that significantly improve the benefit–risk ratio of aminoglycosides which is much needed in the current environment where the rise of antimicrobial resistance poses a major threat to the global population.

## References

[1] Begg EJ, Barclay ML (1995). Aminoglycosides—50 years on. Br J Clin Pharmacol.

[2] Mingeot-Leclercq MP, Tulkens PM (1999). Aminoglycosides: Nephrotoxicity. Antimicrob Agents Chemother.

[3] Schmitz C, Hilpert J, Jacobsen C, Boensch C, Christensen EI, Luft FC, Willnow TE (2002). Megalin deficiency offers protection from renal aminoglycoside accumulation. J Biol Chem.

[4] Christensen EI, Birn H (2001). Megalin and cubilin: synergistic endocytic receptors in renal proximal tubule. Am J Physiol Renal Physiol.

[5] Antoine DJ, Srivastava A, Pirmohamed M, Park BK (2010). Statins inhibit aminoglycoside accumulation and cytotoxicity to renal proximal tubule cells. Biochem Pharmacol.

[6] Taber SS, Pasko DA (2008). The epidemiology of drug-induced disorders: The kidney. Expert Opin Drug Saf.

[7] Silverblatt FJ, Kuehn C (1979). Autoradiography of gentamicin uptake by the rat proximal tubule cell. Kidney Int.

[8] Lopez-Novoa JM, Quiros Y, Vicente L, Morales AI, Lopez-Hernandez FJ (2011). New insights into the mechanism of aminoglycoside nephrotoxicity: an integrative point of view. Kidney Int.

[9] Regec AL, Trump BF, Trifilis AL (1989). Effect of gentamicin on the lysosomal system of cultured human proximal tubular cells. Endocytotic activity, lysosomal pH and membrane fragility. Biochem Pharmacol.

[10] Servais H, Van Der Smissen P, Thirion G, Van der Essen G, Van BF, Tulkens PM, Mingeot-Leclercq MP (2005). Gentamicin-induced apoptosis in LLC-PK1 cells: involvement of lysosomes and mitochondria. Toxicol Appl Pharmacol.

[11] Chwieralski CE, Welte T, Bühling F (2006). Cathepsin-regulated apoptosis. Apoptosis.

[12] Golstein P, Kroemer G (2007). Cell death by necrosis: towards a molecular definition. Trends Biochem Sci.

[13] Peyrou M, Hanna PE, Cribb AE (2007). Cisplatin, gentamicin, and p-aminophenol induce markers of endoplasmic reticulum stress in the rat kidneys. Toxicol Sci.

[14] Usami SI, Abe S, Shinkawa H, Kimberling WJ (1998). Sensorineural hearing loss caused by mitochondrial dna mutations: Special reference to the A1555G mutation. J Commun Disord.

[15] Raggi C, Fujiwara K, Leal T, Jouret F, Devuyst O, Terryn S (2011). Decreased renal accumulation of aminoglycoside reflects defective receptor-mediated endocytosis in cystic fibrosis and Dent’s disease. Pflugers Arch.

[16] Akcan-Arikan A, Zappitelli M, Loftis LL, Washburn KK, Jefferson LS, Goldstein SL (2007). Modified RIFLE criteria in critically ill children with acute kidney injury. Kidney Int.

[17] Moffett BS, Goldstein SL (2011). Acute kidney injury and increasing nephrotoxic-medication exposure in noncritically-ill children. Clin J Am Soc Nephrol.

[18] Mehta RL, Kellum JA, Shah SV, Molitoris BA, Ronco C, Warnock DG, Levin A (2007). Acute Kidney Injury Network: report of an initiative to improve outcomes in acute kidney injury. Crit Care.

[19] Zappitelli M, Moffett BS, Hyder A, Goldstein SL (2011). Acute kidney injury in non-critically ill children treated with aminoglycoside antibiotics in a tertiary healthcare centre: a retrospective cohort study. Nephrol Dial Transplant.

[20] Zappitelli M, Parikh CR, Akcan-Arikan A, Washburn KK, Moffett BS, Goldstein SL (2008). Ascertainment and epidemiology of acute kidney injury varies with definition interpretation. Clin J Am Soc Nephrol.

[21] Kellum JA, Lameire N, Aspelin P, Barsoum RS, Burdmann EA, Goldstein SL, Herzog CA, Joannidis M, Kribben A, Levey AS, MacLeod AM, Mehta RL, Murray PT, Naicker S, Opal SM, Schaefer F, Schetz M, Uchino S (2012). Kidney disease: Improving global outcomes (KDIGO) acute kidney injury work group. KDIGO clinical practice guideline for acute kidney injury. Kidney Int Suppl.

[22] Mehta RL, Awdishu L, Davenport A, Murray PT, Macedo E, Cerda J, Chakaravarthi R, Holden AL, Goldstein SL (2015). Phenotype standardization for drug-induced kidney disease. Kidney Int.

[23] Pirmohamed M, Aithal GP, Behr E, Daly A, Roden D (2011). The phenotype standardization project: Improving pharmacogenetic studies of serious adverse drug reactions. Clin Pharmacol Ther.

[24] Gallagher RM, Kirkham JJ, Mason JR, Bird KA, Williamson PR, Nunn AJ, Turner MA, Smyth RL, Pirmohamed M (2011). Development and inter-rater reliability of the Liverpool adverse drug reaction causality assessment tool. PLoS ONE.

[25] Goldstein SL, Kirkendall E, Nguyen H, Schaffzin JK, Bucuvalas J, Bracke T, Seid M, Ashby M, Foertmeyer N, Brunner L, Lesko A, Barclay C, Lannon C, Muething S (2013). Electronic health record identification of nephrotoxin exposure and associated acute kidney injury. Pediatrics.

[26] Turner MA, Lewis S, Hawcutt DB, Field D (2009). Prioritising neonatal medicines research: UK Medicines for Children Research Network scoping survey. BMC Pediatr.

[27] Clark RH, Bloom BT, Spitzer AR, Gerstmann DR (2006). Reported medication use in the neonatal intensive care unit: Data from a large national data set. Pediatrics.

[28] National Institute of Health and Care Excellence (2012) Antibiotics for early onset neonatal infection. CG149. National Institute for Health and Care Excellence, London. https://guidance.nice.org.uk/cg149. Accessed 28 Sept 2016

[29] Nestaas E, Bangstad HJ, Sandvik L, Wathne KO (2005). Aminoglycoside extended interval dosing in neonates is safe and effective: a meta-analysis. Arch Dis Child Fetal Neonatal Ed.

[30] Rhone ET, Carmody JB, Swanson JR, Charlton JR (2014). Nephrotoxic medication exposure in very low birth weight infants. J Matern Fetal Neonatal Med.

[31] Kent A, Turner MA, Sharland M, Heath PT (2014). Aminoglycoside toxicity in neonates: Something to worry about?. Expert Rev Anti Infect Ther.

[32] Martínková J, Pokorná P, Záhora J, Chládek J, Vobruba V, Selke-Krulichová I, Chládková J (2010). Tolerability and outcomes of kinetically guided therapy with gentamicin in critically ill neonates during the first week of life: an open-label, prospective study. Clin Ther.

[33] Cystic Fibrosis Trust (2011) UK CF Registry Annual Data Report 2009. Cystic Fibrosis Trust, Bromley Kent

[34] Bertenshaw C, Watson AR, Lewis S, Smyth A (2007). Survey of acute renal failure in patients with cystic fibrosis in the UK. Thorax.

[35] Smyth A, Lewis S, Bertenshaw C, Choonara I, McGaw J, Watson A (2008). Case-control study of acute renal failure in patients with cystic fibrosis in the UK. Thorax.

[36] Downes KJ, Rao MB, Kahill L, Nguyen H, Clancy JP, Goldstein SL (2014). Daily serum creatinine monitoring promotes earlier detection of acute kidney injury in children and adolescents with cystic fibrosis. J Cyst Fibros.

[37] Downes KJ, Patil NR, Rao MB, Koralkar R, Harris WT, Clancy JP, Goldstein SL, Askenazi DJ (2015). Risk factors for acute kidney injury during aminoglycoside therapy in patients with cystic fibrosis. Pediatr Nephrol.

[38] Menon S, Kirkendall ES, Nguyen H, Goldstein SL (2014). Acute kidney injury associated with high nephrotoxic medication exposure leads to chronic kidney disease after 6 months. J Pediatr.

[39] Al-Aloul M, Miller H, Alapati S, Stockton PA, Ledson MJ, Walshaw MJ (2005). Renal impairment in cystic fibrosis patients due to repeated intravenous aminoglycoside use. Pediatr Pulmonol.

[40] Pedersen SS, Jensen T, Osterhammel D, Osterhammel P (1987). Cumulative and acute toxicity of repeated high-dose tobramycin treatment in cystic fibrosis. Antimicrob Agents Chemother.

[41] Askenazi DJ, Ambalavanan N, Goldstein SL (2009). Acute kidney injury in critically ill newborns: what do we know? What do we need to learn?. Pediatr Nephrol.

[42] Waikar SS, Bonventre JV (2009). Creatinine kinetics and the definition of acute kidney injury. J Am Soc Nephrol.

[43] Dieterle F, Sistare F, Goodsaid F, Papaluca M, Ozer JS, Webb CP, Baer W, Senagore A, Schipper MJ, Vonderscher J, Sultana S, Gerhold DL, Phillips JA, Maurer G, Carl K, Laurie D, Harpur E, Sonee M, Ennulat D, Holder D, Andrews-cleavenger D, Y-z G, Thompson KL, Goering PL, J-m V, Abadie E, Maciulaitis R, Jacobson-kram D, Defelice AF, Hausner EA, Blank M, Thompson A, Harlow P, Throckmorton D, Xiao S, Xu N, Taylor W, Vamvakas S, Flamion B, Lima BS, Kasper P, Pasanen M, Prasad K, Troth S, Bounous D, Robinson-gravatt D, Betton G, Davis MA, Akunda J, McDuffie JE, Suter L, Obert L, Guffroy M, Pinches M, Jayadev S, Blomme EA, Beushausen SA, Barlow VG, Collins N, Waring J, Honor D, Snook S, Lee J, Rossi P, Walker E, Mattes W (2010). Renal biomarker qualification submission: a dialog between the FDA–EMEA and Predictive Safety Testing Consortium. Nat Biotechnol.

[44] Bonventre JV, Vaidya VS, Schmouder R, Feig P, Dieterle F (2010). Next-generation biomarkers for detecting kidney toxicity. Nat Biotechnol.

[45] Yu Y, Jin H, Holder D, Ozer JS, Villarreal S, Shughrue P, Shi S, Figueroa DJ, Clouse H, Su M, Muniappa N, Troth SP, Bailey W, Seng J, Aslamkhan AG, Thudium D, Sistare FD, Gerhold DL (2010). Urinary biomarkers trefoil factor 3 and albumin enable early detection of kidney tubular injury. Nat Biotechnol.

[46] Vaidya VS, Ozer JS, Dieterle F, Collings FB, Ramirez V, Troth S, Muniappa N, Thudium D, Gerhold D, Holder DJ, Bobadilla NA, Marrer E, Perentes E, Cordier A, Vonderscher J, Maurer G, Goering PL, Sistare FD, Bonventre JV (2010). Kidney injury molecule-1 outperforms traditional biomarkers of kidney injury in preclinical biomarker qualification studies. Nat Biotechnol.

[47] McWilliam SJ, Antoine DJ, Sabbisetti V, Turner MA, Farragher T, Bonventre JV, Park BK, Smyth RL, Pirmohamed M (2012). Mechanism-based urinary biomarkers to identify the potential for aminoglycoside-induced nephrotoxicity in premature neonates: A proof-of-concept study. PLoS ONE.

[48] Lahiri T, Guillet A, Diehl S, Ferguson M (2014). High-dose ibuprofen is not associated with increased biomarkers of kidney injury in patients with cystic fibrosis. Pediatr Pulmonol.

[49] McWilliam SJ, Antoine DJ, Smyth RL, Pirmohamed M (2014). 66 Association of urinary kidney injury molecule-1 with aminoglycoside exposure in children with cystic fibrosis. J Cyst Fibros.

[50] Uluer AZ, Casey A, Jawaid N, Fowler R, Demars N, Vaidya V, Waikar S, Bonventre JV, Ferguson M (2010). Urinary biomarkers for early detection of nephrotoxicity in cystic fibrosis. Pediatr Pulmonol.

[51] Buring JE, Evans DA, Mayrent SL, Rosner B, Colton T, Hennekens CH (1988). Randomized trials of aminoglycoside antibiotics: quantitative overview. Rev Infect Dis.

[52] Pitt TL, Sparrow M, Warner M, Stefanidou M (2003). Survey of resistance of Pseudomonas aeruginosa from UK patients with cystic fibrosis to six commonly prescribed antimicrobial agents. Thorax.

[53] Bockenhauer D, Hug MJ, Kleta R (2009). Cystic fibrosis, aminoglycoside treatment and acute renal failure: The not so gentle micin. Pediatr Nephrol.

[54] Smyth A, Tan KHV, Hyman-Taylor P, Mulheran M, Lewis S, Stableforth D, Knox A (2005). Once versus three-times daily regimens of tobramycin treatment for pulmonary exacerbations of cystic fibrosis—the TOPIC study: a randomised controlled trial. Lancet.

[55] Knoderer CA, Nichols KR, Cox EG (2014). Optimized antimicrobial dosing strategies: a survey of pediatric hospitals. Pediatr Drugs.

[56] Contopoulos-Ioannidis DG, Giotis ND, Baliatsa DV, Ioannidis JP (2004). Extended-interval aminoglycoside administration for children: a meta-analysis. Pediatrics.

[57] UK CF Trust Antibiotic Working Group (2009) Antibiotic treatment for cystic fibrosis. Cystic Fibrosis Trust. Bromley Kent. https://www.cysticfibrosis.org.uk/~/media/documents/the-work-we-do/care/consensus-documents/antibiotic-treatment-cystic-fibrosis-may-09.ashx?la=en. Accessed 28 Sept 2016

[58] Flume PA, Mogayzel PJ, Robinson KA, Goss CH, Rosenblatt RL, Kuhn RJ, Marshall BC, Bujan J, Downs A, Finder J, Goss C, Gutierrez H, Hazle L, Lester M, Quittell L, Sabadosa K, Vender RL, White TB, Willey-Courand DB, Saldanha I, Oyegunle M, Shankar MB, McKoy N, Sengupta S, Odelola OA, Waybright S (2009). Cystic fibrosis pulmonary guidelines: Treatment of pulmonary exacerbations. Am J Respir Crit Care Med.

[59] Coulthard KP, Peckham DG, Conway SP, Smith CA, Bell J, Turnidge J (2007). Therapeutic drug monitoring of once daily tobramycin in cystic fibrosis—caution with trough concentrations. J Cyst Fibros.

[60] Ryan G, Singh M, Dwan K (2011). Inhaled antibiotics for long-term therapy in cystic fibrosis. Cochrane Database Syst Rev.

[61] Hennig S, McKay K, Vidmar S, O’Brien K, Stacey S, Cheney J, Wainwright CE (2014). Safety of inhaled (Tobi®) and intravenous tobramycin in young children with cystic fibrosis. J Cyst Fibros.

[62] Hoffmann IM, Rubin BK, Iskandar SS, Schechter MS, Nagaraj SK, Bitzan MM (2002). Acute renal failure in cystic fibrosis: association with inhaled tobramycin therapy. Pediatr Pulmonol.

[63] Goldstein SL, Mottes T, Simpson K, Barclay C, Muething S, Haslam DB, Kirkendall ES (2016). A sustained quality improvement program reduces nephrotoxic medication-associated acute kidney injury. Kidney Int.

[64] Prayle AP, Jain K, Touw DJ, Koch BCP, Knox AJ, Watson A, Smyth AR (2016). The pharmacokinetics and toxicity of morning vs. evening tobramycin dosing for pulmonary exacerbations of cystic fibrosis: A randomised comparison. J Cyst Fibros.

[65] Balakumar P, Rohilla A, Thangathirupathi A (2010). Gentamicin-induced nephrotoxicity: Do we have a promising therapeutic approach to blunt it?. Pharmacol Res.

[66] Vlašiæ-Matas J, Rumboldt Z, Kareloviæ D (2000). Renoprotective role of nifedipine during gentamicin therapy: Randomized controlled trial. Croat Med J.

[67] Al-Aloul M, Miller H, Ledson MJ, Walshaw MJ (2004). Renoprotective effect of fosfomycin in the treatment of multiresistant Pseugomonas aeruginosa in CF. Thorax.

[68] Watanabe A, Nagai J, Adachi Y, Katsube T, Kitahara Y, Murakami T, Takano M (2004). Targeted prevention of renal accumulation and toxicity of gentamicin by aminoglycoside binding receptor antagonists. J Control Release.

[69] Verhulst A, D’Haese PC, De Broe ME (2004). Inhibitors of HMG-CoA reductase reduce receptor-mediated endocytosis in human kidney proximal tubular cells. J Am Soc Nephrol.

[70] Sidaway JE, Davidson RG, McTaggart F, Orton TC, Scott RC, Smith GJ, Brunskill NJ (2004). Inhibitors of 3-hydroxy-3-methylglutaryl-CoA reductase reduce receptor-mediated endocytosis in opossum kidney cells. J Am Soc Nephrol.

[71] Chinnapa Reddy V, Amulya V, Anusha Lakshmi C, Bala Praveen Kumar Reddy D, Pratima D, Thanga Thirupathi A, Pavan Kumar K, Sengottuvelu S (2012). Effect of simvastatin in gentamicin induced nephrotoxicity in albino rats. Asian J Pharm Clin Res.

[72] Jabbari M, Rostami Z, Jenabi A, Bahrami A, Mooraki A (2011). Simvastatin ameliorates gentamicin-induced renal injury in rats. Saudi J Kidney Dis Transpl.

[73] Zhang J, Brown RP, Shaw M, Vaidya VS, Zhou Y, Espandiari P, Sadrieh N, Stratmeyer M, Keenan J, Kilty CG, Bonventre JV, Goering PL (2008). Immunolocalization of Kim-1, RPA-1, and RPA-2 in kidney of gentamicin-, mercury-, or chromium-treated rats: relationship to renal distributions of iNOS and nitrotyrosine. Toxicol Pathol.

[74] Ichimura T, Asseldonk EJPV, Humphreys BD, Gunaratnam L, Duffield JS, Bonventre JV (2008). Kidney injury molecule-1 is a phosphatidylserine receptor that confers a phagocytic phenotype on epithelial cells. J Clin Invest.

[75] Coca SG, Yalavarthy R, Concato J, Parikh CR (2008). Biomarkers for the diagnosis and risk stratification of acute kidney injury: a systematic review. Kidney Int.

[76] Kjeldsen L, Johnsen AH, Sengelov H, Borregaard N (1993). Isolation and primary structure of NGAL, a novel protein associated with human neutrophil gelatinase. J Biol Chem.

[77] Mishra J, Mori K, Ma Q, Kelly C, Barasch J, Devarajan P (2004). Neutrophil gelatinase-associated lipocalin: A novel early urinary biomarker for cisplatin nephrotoxicity. Am J Nephrol.

[78] Suchojad A, Tarko A, Smertka M, Majcherczyk M, Brzozowska A, Wroblewska J, Maruniak-Chudek I (2015). Factors limiting usefulness of serum and urinary NGAL as a marker of acute kidney injury in preterm newborns. Ren Fail.

[79] Skálová S (2005). The diagnostic role of urinary N-acetyl-beta-D-glucosaminidase (NAG) activity in the detection of renal tubular impairment. Acta Med (Hradec Kralove).

[80] Price RG (1992). The role of NAG (N-acetyl-β-D-glucosaminidase) in the diagnosis of kidney disease including the monitoring of nephrotoxicity. Clin Nephrol.

